# Enhanced Immunomodulatory Activity of Gelatin-Encapsulated *Rubus coreanus* Miquel Nanoparticles

**DOI:** 10.3390/ijms12129031

**Published:** 2011-12-07

**Authors:** Yong Chang Seo, Woon Yong Choi, Choon Geun Lee, Seon Woo Cha, Young Ock Kim, Jin-Chul Kim, Gregor P. C. Drummen, Hyeon Yong Lee

**Affiliations:** 1 Department of Biomaterials Engineering, Kangwon National University, Chuncheon 200-701, Korea; E-Mails: yongchang2da@kangwon.ac.kr (Y.C.S.); cwy1012@kangwon.ac.kr (W.Y.C.); lek6919@kangwon.ac.kr (C.G.L); jinkim@kangwon.ac.kr (J.-C.K.); 2 Medical & Bio-material Research Center, Kangwon National University, Chuncheon 200-701, Korea; 3 Department of Herbal Crop Research, National Institute of Horticultural & Herbal Science, RDA, Eumseong 369-873, Korea; E-Mails: chasw@rda.go.kr (S.W.C.); kyo9128@korea.kr (Y.O.K.); 4 Bionanoscience and Bio-Imaging Program, Cellular Stress and Ageing Program, Bio&Nano-Solutions, D-40472 Düsseldorf, Germany

**Keywords:** *Rubus coreanus* Miquel, nano-encapsulation process, nanoparticle, immune activity, immunomodulation, edible polymer, gelatin, ferulic acid, cytotoxicity

## Abstract

The aim of this work was to investigate the immunomodulatory activities of *Rubus coreanus* Miquel extract-loaded gelatin nanoparticles. The mean size of the produced nanoparticles was 143 ± 18 nm with a bandwidth of 76 nm in the size distribution and a maximum size of ~200 nm, which allows effective nanoparticle uptake by cells. Confocal imaging confirmed this, since the nanoparticles were internalized within 30 min and heterogeneously distributed throughout the cell. Zeta-potential measurements showed that from pH = 5 onwards, the nanoparticles were highly negatively charged, which prevents agglomeration to clusters by electrostatic repulsion. This was confirmed by TEM imaging, which showed a well dispersed colloidal solution. The encapsulation efficiency was nearly 60%, which is higher than for other components encapsulated in gelatin nanoparticles. Measurements of immune modulation in immune cells showed a significant effect by the crude extract, which was only topped by the nanoparticles containing the extract. Proliferation of B-, T- and NK cells was notably enhanced by *Rubus coreanus*-gelatin nanoparticles and in general ~2–3 times higher than control and on average ~2 times higher than ferulic acid. *R. coreanus*-gelatin nanoparticles induced cytokine secretion (IL-6 and TNF-α) from B- and T-cells on average at a ~2–3 times higher rate compared with the extract and ferulic acid. *In vivo* immunomodulatory activity in mice fed with *R. coreanus*-gelatin nanoparticles at 1 mL/g body weight showed a ~5 times higher antibody production compared to control, a ~1.3 times higher production compared to the extract only, and a ~1.6 times higher production compared to ferulic acid. Overall, our results suggest that gelatin nanoparticles represent an excellent transport vehicle for *Rubus coreanus* extract and extracts from other plants generally used in traditional Asian medicine. Such nanoparticles ensure a high local concentration that results in enhancement of immune cell activities, including proliferation, cytokine secretion, and antibody production.

## 1. Introduction

Biodegradable nanoparticles have been used frequently as drug delivery vehicles due to their high bioavailability, good encapsulation properties, and relative lack of toxicity [1]. As the basis for a natural encapsulation agent, gelatin is widely used in a number of parenteral formulations because of its biocompatibility [2], biodegradability [3], and low antigenicity [4]. Gelatin, which is a mixture of soluble proteins and peptides, is obtained by partial hydrolysis of collagen, the main fibrous protein constituent in bones, cartilage, and skin [5]. Furthermore, because gelatin is a complex poly-ampholyte with cross-linking properties that depend significantly on temperature and on cationic, anionic, and hydrophilic groups [6], it has been commonly used as a biomaterial in the manufacturing of food and cosmetic products, allowing significant control over the final product. In addition, the protein structure of the constituents is well defined, offering a large number of functional groups for further derivatization. Because of its favorable and biocompatible properties, gelatin is classified as a food product and as such is not assigned an E-number [7]. In pharmaceuticals, gelatin is typically used in the shells of capsules to make the powdery content easier to transport by ingestion or subcutaneous injection [8] and numerous drugs and even engineered nanoparticles have successfully been encapsulated in gelatin nanoparticles or coated with gelatin, with efficient loading and drug release properties [8–10]. Furthermore, because of these advantages, the technology of nano-encapsulation has been extended to natural products over the past decade to protect them from chemical damage and product degradation, especially from air oxidation, and therefore extent the product’s shelf-life before its final application [11].

Components from edible plants, e.g., fruits and vegetables, generally termed phytochemicals, have draw increased attention because of their potential benefits to human health [12]. It has long been known, since medieval times, that humans inherently depend on nutritional intake from plants to cover their need for vitamins, antioxidants, minerals, and trace elements to name but a few. In those days, prolonged exploratory travels and military campaigns caused scurvy (*syn*. scorbut), a vitamin C (ascorbate) deficiency that led to formation of spots on the skin, spongy gums, and bleeding from the mucous membranes [13], which impressively exemplifies the importance of plants as a source of essential phytochemicals. Furthermore, herbs and plants have been used in experience-based medical practice both in the Occident and Orient for many centuries. However, in Asian countries, the use of medicinal plants in traditional medical practice has a much longer history and is more pronounced. Not surprisingly, interest in traditional Asian medicine has increased significantly over the past few decades including in the West, because of the recognized potential of developing new drugs based on natural phytochemicals.

*Rubus coreanus* Miquel or Korean black raspberry is a perennial shrub belonging to the *Rosaceae* family that produces edible berries and is predominantly endogenous to the southern part of Korea and parts of China and Japan. The incomplete ripened fruit has been used in traditional herbal medicine, since anti-impotence, aphrodisiacal, anti-inflammatory [14], anti-bacterial [15], and antioxidative properties [16] are ascribe to *R. coreanus*. In addition, it is an effective agent against allergic diseases [17] and colon cancer [18].

Because so many bioremedial effects have previously been reported both by traditional practitioners and researchers and because most of the effects in one way or another involve components of the immune system, we aimed with the current study to investigate the immunomodulatory activities of *Rubus coreanus* Miquel extract-loaded gelatin nanoparticles. Furthermore, since delivery vehicles are crucial in drug formulation, stability, bioavailability, and uptake, we assessed whether gelatin nanoparticles would provide a cheap, biocompatible, low toxic and convenient way to introduce the extracts orally, with the possible future option to target the nanoparticles to particular tissues and achieve a high local concentration and bioremedial effect. In this study, gelatin nanoparticles in a narrow size range were prepared by ultrasonic treatment of aqueous *R. coreanus* extracts. The nanoparticles of *R. coreanus* were characterized by various physicochemical means, such as size measurements, loading capacity, cytotoxicity assessment, transmission electron microscopic (TEM) evaluation and several methods to assess their immune modulatory activities.

Our results show that *R. coreanus* extract-encapsulated gelatin nanoparticles with an average size of 143 ± 18 nm and a narrow bandwidth can effectively and easily be produced. This offers the possibility to add targeting sequencings to target such nanoparticles to particular tissues and as such achieve a higher local concentration compared to ingestion of the crude extract only. Toxicity assessment shows a low toxicity and high biocompatibility. Immunomodulatory effects were determined in T-, B-, and NK-cells and in all cell types, *R. coreanus* extract-encapsulated gelatin nanoparticles showed a higher modulatory potency compared with the crude extract or ferulic acid as a control. Confocal imaging showed that the loaded nanoparticles were taken-up within 30 min and were distributed homogenously throughout the cell.

## 2. Results and Discussion

### 2.1. Characterization of Nanoparticles

#### 2.1.1. Size and Morphology of Nanoparticles

**Figure 1:**
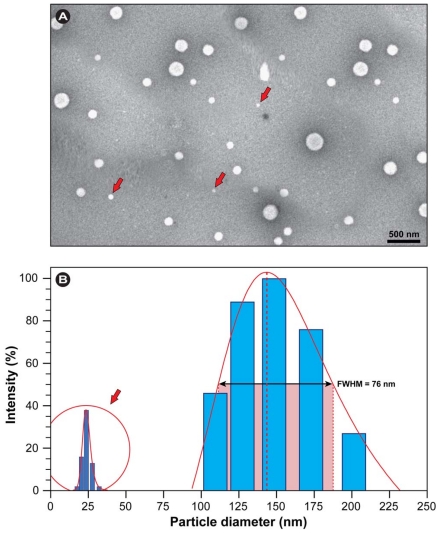
Analysis of nanoparticle morphology and size distribution of gelatin nanoparticles containing *R. coreanus* extract. (**A**) TEM: red arrows indicate nanoparticles in the 25 nm range belonging to the small distribution peak; (**B**) Size distribution and Gaussian fit after DLS analysis.

Gelatin nanoparticles containing *R. coreanus* (GNR) were characterized through transmission electron microscopy (TEM) to evaluate the morphology of the individual particles and the mean particle size and size distribution was assessed via dynamic light scattering (DLS). The results in Figure 1(A) show that the particles formed were of spherical shape and that predominantly two different pools of particles were formed. This was corroborated by the DLS measurements, which show a pool of smaller particles (red arrows) with an average diameter of 23 ± 6 nm and narrow band width and the majority as larger particles with an average diameter of 143 ± 18 nm and a bandwidth of 76 nm.

The size distribution, as deduced from the full-width-at-half-maximum (FWHM), is sufficiently narrow and the maximum size of approximately 200 nm ensures good cellular penetration and uptake via endocytic and passive mechanisms [19,20]. Notice that the nanoparticles observed present themselves as white domains because the samples were pretreated by a negative staining technique. No noteworthy nanoparticles-clusters were observed, indicating a good colloidal stability.

In general, the TEM image of GNR shows a uniform carrier system with two different size pools and the results are largely in agreement with observations by others [21]. Li *et al*. [22] recently produced self-assembling, amphiphilically-modified gelatin nanoparticles in which the size could be controlled by hydrophobic substitution. They observed good cellular uptake with gelatin nanoparticles up to 130 nm. Therefore, the combined results show that based on their size and surface properties, gelatin nanoparticles constitute ideal vehicles for extracts of phytochemicals in biomedical applications.

#### 2.1.2. Zeta Potential of Nanoparticles

**Figure 2:**
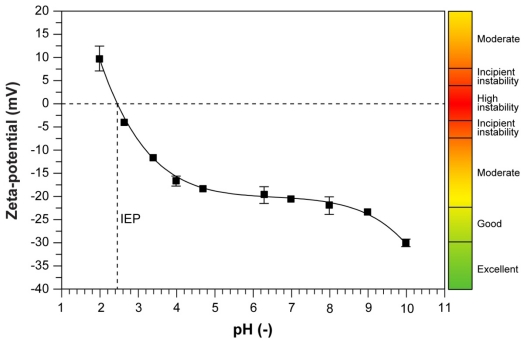
Zeta potential of gelatin nanoparticles containing *R. coreanus* extract.

In order to assess the stability of the colloidal gelatin nanoparticles in solutions, zeta potential measurements were preformed. Figure 2 shows the pH-dependent zeta potentials of GNRs. The absolute value of the zeta potential decreased with pH, *i.e.*, the zeta potential was 9.6 mV at pH = 2, −18.2 mV at pH = 6.15 and −28.6 mV at pH = 10. Most importantly, at physiological pH = 7.4, the average zeta potential of GNR was −19.3 mV. The measurements show that GNR nanoparticles have a good stability over a wide pH range from approximately pH = 4 to 10 as reflected by the high negative charges that prevent agglomeration by inter-particulate electrostatic repulsion forces [23]. This might be expected, since gelatin is a heterogeneous mixture of single or multi-stranded polypeptides, predominantly consisting of the acidic and basic amino acids arginine (7.8%) and glutamic acid (10%), the hydrophobic alanine (8.9%), and the zwitter ionic, glycine (21%), proline, and hydroxyproline (both 12%). Because of the inductive effect by the carbonyls (electron-withdrawing) and the lone pairs on the nitrogen of the amide groups, resonance charge delocalization leads to a net negative, positive or neutral charge in the gelatin chain depending on the pH of the solution [24]. With a measured isolectric point around 5.0 (4.84 [25]; 4.88 [26]) in pure gelatin nanoparticles, at neutral to basic pH values, the carboxylic groups are deprotonized, and an overall net negative electric potential in the interfacial double layer is measured. These results are in good agreement with the TEM observations and other reports in the literature [22,24]. However, as shown in Figure 2, the isoelectric point of GNRs significantly shifts to lower values when the extract components are present, *i.e.*, 2.48 ± 0.20.

Furthermore, it was recently reported that nanoparticle-based colloidal solutions are inherently more stable compared to micrometer-sized particle suspensions, because of the much higher Brownian motion of suspended nanoparticles that at least partially and mechanically prevents agglomeration [27].

#### 2.1.3. Encapsulation Efficiency

**Figure 3:**
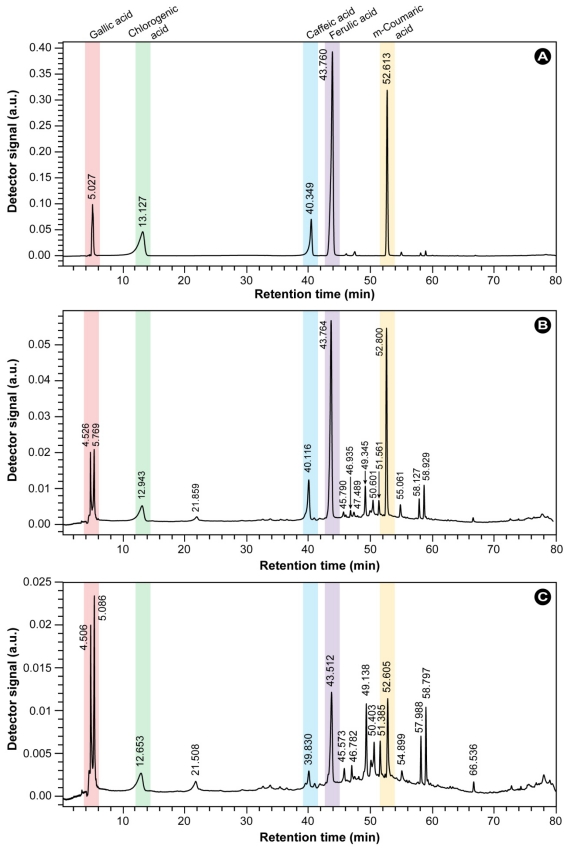
Chromatograms of (**A**) five phenolic acid standards; (**B**) crude extract of *R. coreanus* (added initially); and (**C**) *R. coreanus* extract-loaded GNR nanoparticles extracted by chemical treatment.

To determine the encapsulation efficiency of the *R. coreanus* extracts in gelatin nanoparticles, high-performance liquid chromatography (HPLC) analysis was performed, which additionally provided an initial assessment of the extract’s composition. Figure 3(A) shows representative HPLC chromatograms for five phenolic acid standards, *i.e.*, gallic acid, chlorogenic acid, caffeic acid, ferulic acid, and m-coumaric acid. In Figures 3(B,C), the chromatograms of the *R. coreanus* crude extract and *R. coreanus* extract-loaded GNR, which were chemically treated to release their content, are shown. Comparison of the peak positions revealed that the extract is rich in chlorogenic acid, caffeic acid, ferulic acid, and m-coumaric acid. The figure also shows that in addition to gallic acid, a derivative is present with a slightly different retention time; the nature of the split peak at ~5 min and the unidentified minor peaks in the chromatograms is currently under investigation.

The overall encapsulation efficiency of the *R. coreanus* extracts in GNRs was close to 60% and this was significantly higher than the entrapment ratio of conventional gelatin nanoparticles (<45%) as reported by Saxena [21] and Vandervoort [28] and their respective co-workers. From the HPLC analysis, the encapsulation efficiency of the major components in GNR nanoparticles were determined to be 70.6% for gallic acid, 72.1% for chlorogenic acid, 63.2% for caffeic acid, 44.4% for ferulic acid and 45.8% for m-coumaric acid. These results were similar to the encapsulation efficiency of other water-soluble active substances [29]. Based on these results, it may be concluded that gelatin is a suitable carrier for the encapsulation of phytochemical extracts.

### 2.2. Toxicity Assessment

#### 2.2.1. *In Vitro* Cytotoxicity

**Figure 4:**
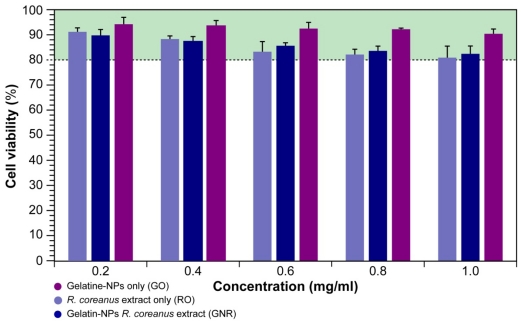
Cytotoxicity assessment in HEK293 cells after 48 h incubation with gelatin nanoparticles containing *R. coreanus* extract or the extract only compared to gelatin nanoparticles as a control.

Cytotoxicity evaluation was performed with the sulforhodamine B (SRB) assay in HEK293 cells, which are human embryonic kidney cells and a good model for such evaluations. The data are expressed as percentage viable cells. As shown in Figure 4, gelatin nanoparticles only (GO) show maximally a 10% decrease in cell viability at the highest concentration (1 mg/mL) after 48 h incubation. Both the addition of GNR and the extract only (RO) result in a concentration-dependent decrease in cell viability to a maximum of 17.6 and 19.2% respectively. The figure also illustrates that there is virtually no significant difference in toxicity between the encapsulated and extract forms. Furthermore, the difference in cytotoxicity compared to the GO is practically negligible.

A number of studies using modified gelatin nanoparticles, including inorganic nanoparticles coated with gelatin report similar results [22,30]. Furthermore, comparison of the cytotoxic effects of extracts from other medicinal plants, such as the traditional Chinese medicinal plant *Ligularia hodgsonii* Hook, which contains pyrrolizidine alkaloids such as clivorine, puts our results further into perspective. This plant is traditionally used to treat cough, hepatitis, and inflammation and shows a reduction of HEK293 cell viability to 60% after treatment with 100 μM of the active component for 48 h [31], which is a concentration that is 24 times less than the maximum concentration used in our study.

In general, our results are in accordance with other studies [22,30] and show that HEK293 cells cultured in the presence of GNR for 48 h remain viable over a wide concentration range showing only a minor cytotoxic effect in a concentration-dependent manner. Nonetheless, for further and safe *in vivo* animal studies and clinical research on extracts from medicinal plants used in traditional Asian medicine, assessment of cytotoxicity *in cellulo* does not suffice and further studies in intact organisms are necessary. Therefore, we performed a 21 day assessment in mice (*vide infra*).

#### 2.2.2. *In Vivo* Toxicity

As stated previously, toxicity assessment in intact animals was performed to further determine the safety of GNR, primarily for use in future animal studies. Five-week-old female ICR mice were placed on a 21 day feeding regiment (1 mL/g body weight) for a number of controls and GO or GNR (1 mg/mL), and subsequently body weight, cholesterol and glucose were assessed.

**Figure 5:**
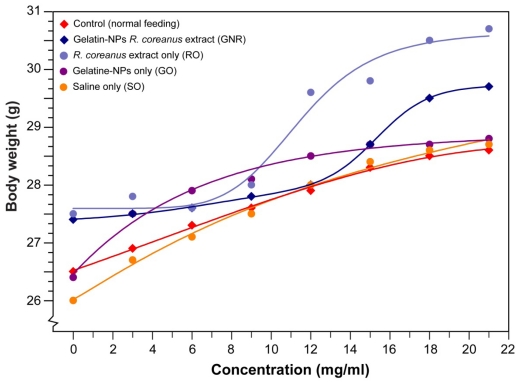
Changes in body weight in female ICR mice fed (1 mL/g body weight) on gelatin nanoparticles with *R. coreanus* extract, gelatin nanoparticles only or *R. coreanus* extract only (1 mg/mL). Normal feeding and saline only were additionally used as controls (Lines represent non-linear growth curve fitting).

In all groups, the mice survived for the entire 21 day experimental period, which shows that the doses used are non-lethal (survival ratio = 1). The body weight of the mice gradually increased in all three groups, albeit that those animals fed on the extract or GNR showed sigmoid growth curves and the daily weight gain was slightly higher in these groups (Figure 5). A steady gain in weigh indicates that no adverse effects undermine appetite and growth. In the group fed only the *R. coreanus* extract (RO), the body weight increased to 30.7 g after 21 days; this weight was marginally higher than the body weights of 29.7 g in the GNR group and 28.8 g in the GO group.

The extracts of *R. coreanus* generally reduced LDL-cholesterol and glucose levels, and increased HDL-cholesterol levels in virtually all feeding groups (Table 1). However, the effects were markedly larger in the GNR group, as glucose decreased to 208 mg/dL, LDL-cholesterol dropped to 45.4 mg/dL, and HDL-cholesterol increased to 72.5 mg/dL. Since high HDL-cholesterol levels are associated with a lower health risk, such as a decreased incidence in sclerotic plague formation and concomitant cardiovascular disease, these results show the potential beneficial effect of GNR supplementation *in vivo*. Here there is a clear advantage of the GNR nanoparticles over the GO extract.

Under exogenous stress conditions, lipid peroxides and oxidized LDL accumulate in the organism and it has been shown that oxidized LDL-cholesterol is more negatively charged with an increased cytotoxicity [32]. Furthermore, macrophages up-regulate their scavenger receptors in response to oxidized-LDL to enhance the uptake of oxidized-LDL (foam cell formation), to name but one consequence of the immune response to stress [33]. Since GNR boost the function of other immunocytes (*vide infra*), it would be interesting to determine how GNR nanoparticles affect both lipoprotein particles and immunocytes in an atherosclerosis model organism, as well as whether GNR prevent the formation of oxidized LDL through antioxidant activity.

Despite the fact that many questions remain, our preliminary results suggest that treatment with GNR might be useful as an anti-stress factor, which might both be related to GNR’s action in boosting the immune system and the antioxidative properties of several components in the extract.

### 2.3. Immune Activities of Nanoparticles

#### 2.3.1. Proliferation of B- and T-Cells

**Figure 6:**
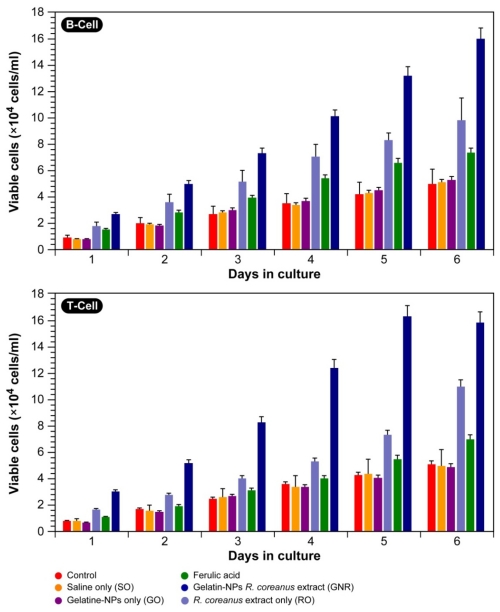
Proliferation of human B- and T-cells cultured in the presence of gelatin nanoparticles containing *R. coreanus* extract and ferulic acid (both 0.5 mg/mL) as a positive control for 6 days.

Recent research suggests that extracts of phytochemicals used in traditional Asian medicine have immunomodulatory properties and contain ingredients that promote immune cell proliferation. Here, it was evaluated if *R. coreanus* extracts might show the same effect and properties. Figure 6 shows the cell counts of human B- and T-cells in samples treated with the extract (0.5 mg/mL) for 6 days; the number of cells increased with time. Ferulic acid, which is a derivative of cinnamic acid that has been reported to have immunomodulatory activity [34], was used as a positive control in the same concentration as the *R. coreanus* extract.

In the controls, normal medium without addition, saline only, gelatin nanoparticles only, cell proliferation progressed normally and near linearly with no significant deviation amongst them. The fact that gelatin generally does not affect cell proliferation was recently established by Magrez *et al.* [35] in human lung-tumor cell line H596 and their results are in agreement with our experiments. Ferulic acid showed a significant induction in proliferation of both T- (~1.25 × control) and B-cells (~1.52 × control), as was to be expected. Both the conventional extract and GNRs increased T- and B-cell proliferation in a time-dependent manner with a deviation from linearity towards an exponential increase, as determined from non-linear curve fitting. Interestingly, the nano-encapsulated extracts induced a significantly higher proliferation in T- (~3.4 × control) and B-cells (~2.9 × control) compared with the extract only, *i.e.*, T- (~1.8 × control) and B-cells (~1.9 × control). This might be an indication that the nanoparticles are better taken-up by the cells and as such a higher intracellular concentration is reached.

Initial experiments by our group [36] previously suggested that raspberries contain components that promote immune cell proliferation and the current results confirm this notion.

#### 2.3.2. Secretion of Cytokines

Cytokines are important signaling molecules that are secreted by immune cells and affect immune system activity *in vivo*. Of the myriad of cytokines that modulate immune cell activity, interleukin-6 (IL-6) and tumor necrosis factor alpha (TNF-α) are important and potent proinflammatory cytokines exerting pleiotropic effects on a number of cell types and are involved in the regulation of the immune response, inflammation, and hematopoiesis [37,38].

**Figure 7:**
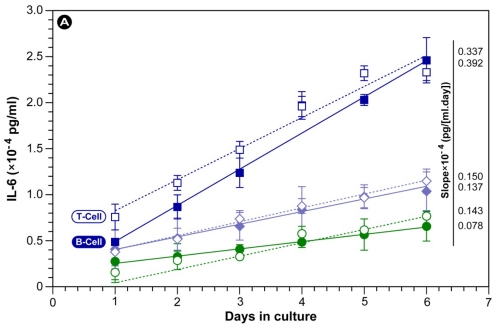

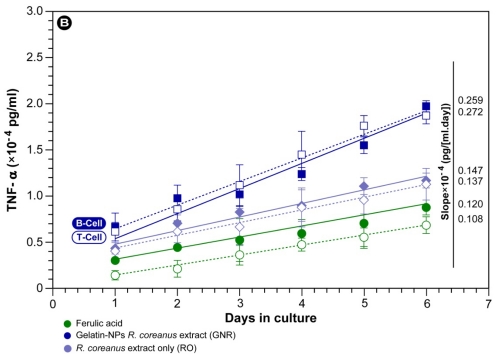
Secretion of IL-6 (**A**) and TNF-α (**B**) from human B- (solid symbols) and T-cells (open symbols) in response to gelatin nanoparticles containing *R. coreanus* extract (GNR), extract only (RO), and ferulic acid as a positive control (0.5 mg/mL).

Figure 7 shows the secretion of the cytokines IL-6 and TNF-α by cultured human B- and T-cells in the presence of the various extracts and ferulic acid over a period of 6 days. The maximal amount of IL-6 and TNF-α released from B-cells grown in the presence of GNRs after this period was 2.46 × 10^−4^ pg/mL and 1.97 × 10^−4^ pg/mL, respectively. Equally, T-cells secreted 2.33 × 10^−4^ pg/mL and 1.87 × 10^−4^ pg/mL of IL-6 and TNF-α maximally, in the presence of GNR. The figure illustrates that both B- and T-cells can be stimulated to produce IL-6 and TNF-α in response to the extract only and GNRs. Furthermore, the fitted slopes in Figure 7 reveal that (i) T- and B-cells are equally stimulated by the samples; (ii) both the extract and the nanoparticles GNR induce the cells more compared with the positive control ferulic acid; (iii) the production of IL-6 is nearly 50% higher than TNF-α; and (iv) on average, GNRs induced cytokine secretion at a ~2–3 times higher rate compared with the extract and ferulic acid; the latter two had secretion rates that were in the same order of magnitude, albeit that the extract caused higher absolute levels.

These secretion values were similar to those reported for the radix extract of *Rosa rugosae* [39]. Thus, the *R. coreanus* extract and the use of nano-encapsulation enhances immune activity by promoting the proliferation of immune cells and increasing the secretion of cytokines. The data in this study also confirm the practical usefulness of GNRs as a functional material associated with immune activation. However, further studies in model animals are required to firmly establish the bioremedial effects on the GNR formulation proposed in this paper. Such studies are currently in preparation.

#### 2.3.3. Proliferation of Natural Killer Cells

Natural killer (NK) cells were first recognized in 1975 for their ability to kill leukemic cells without major-histocompatibility complex (MHC) restriction or prior sensitization [40,41]. They play an important role in immune surveillance and their primary function is to eliminate aberrant cells, including virally infected and tumorigenic cells.

NK cells respond to signaling by interleukins IL-2, IL-15 and IL-21, IL-12 and IL-18 [42,43]. Once activated, NK cells produce both proinflammatory and immunosuppressive cytokines and chemokines, which include TNF-α, IL-10, IL-3, IL-6, interferon gamma (IFN-γ), granulocyte and granulocyte macrophage colony-stimulatory factor (G-CSF and GM-CSF), and growth factor beta (TGF-β) [44]. Recent research shows that NK cells are not only cytolytic effector cells, but exert negative feedback on activated macrophages during microbial infections and act as regulatory cells of cell types, such as dendritic cells, T-cells, B-cells, and endothelial cells. Because NK cells affect other cells of the immune system and link the innate and adaptive immune response, we investigated the effect of the *R. Coreanus* extract and the GNR nanoparticles on NK proliferation as a measure for immunomodulatory properties of the aforementioned species.

**Figure 8:**
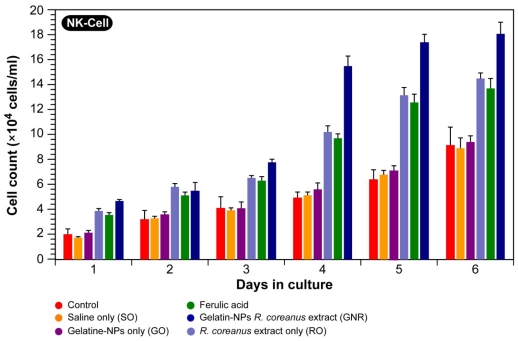
Effects on NK cell proliferation of B-cell supernatants containing 0.5 mg/mL extracts.

The results in Figure 8 show that the NK cell content increased with the addition of the B-cell supernatant containing 0.5 mg/mL of crude *R. coreanus* extract or GNRs and after 6 days in culture, rose to 14.6 × 10^4^ cells/mL or 18.2 × 10^4^ cells/mL for the crude extract and GNR respectively. This corresponds to a ~2–3 times higher proliferation rate in the presence of GNR (Figure 8). Compared with ferulic acid, the progression in NK cell proliferation over the 6 day period in the presence of the crude extract was nearly the same as the positive control, ferulic acid. Furthermore, in the assessment of the NK cell proliferative response to the samples, it is clear that pure gelatin nanoparticles did not induce any effect compared with the control and saline only.

Overall, our results imply that GNRs significantly increase the proliferation of NK cells as compared to control and that the higher effect compared to the crude extracts is likely caused by their cellular penetration and uptake, thereby achieving a higher local concentration.

#### 2.3.4. Antibody Production *in Vivo* Using a Mouse Model

Immunoglobulin G (IgG) is the main protein involved in humoral immunity, contributing substantially to the defense against infection and is the most abundant antibody class in the sera of humans and mice [45]. IgG acts on invading pathogens (“non-self”) by agglutination and immobilization, complement activation via the classical pathway that leads to opsonization and phagocytosis. Thus, the quantification of immunoglobulins in serum is a representative indicator of immune activity [46].

**Figure 9:**
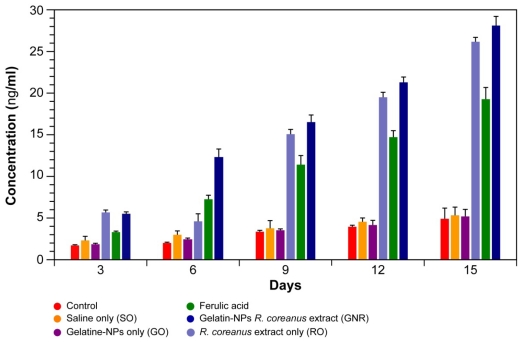
Antibody production *in vivo* in female ICR mice fed gelatin nanoparticles containing *R. coreanus* extract, gelatin nanoparticles only, *R. coreanus* extract only, or ferulic acid (all 1 mg/mL and 1 mL/g body weight).

*In vivo* experiments were conducted with ICR mice over a 15-day period. In total, 46 blood samples obtained at intervals of three days were used to measure IgG antibody production (Figure 9). In all mouse groups, IgG antibody production showed a gradual increase over time. Antibody production increased after the administration of either RO or GNRs. In addition, the antibody production stimulated by RO or GNRs was greater than that stimulated by ferulic acid. The highest antibody concentration was induced by GNRs on day 15, *i.e.*, 28.15 ng/mL. Overall, the GNR group showed a ~5 time higher antibody production compared to control, a ~1.3 time higher production compared to the extract only, and a ~1.6 time higher production compared to ferulic acid (Figure 9). These results further suggest that GNRs might be used as an immune enhancement drug in a myriad of diseases, especially in those in which the immune system is compromised.

### 2.4. Penetration of Nanoparticles into the Immune Cells

**Figure 10:**
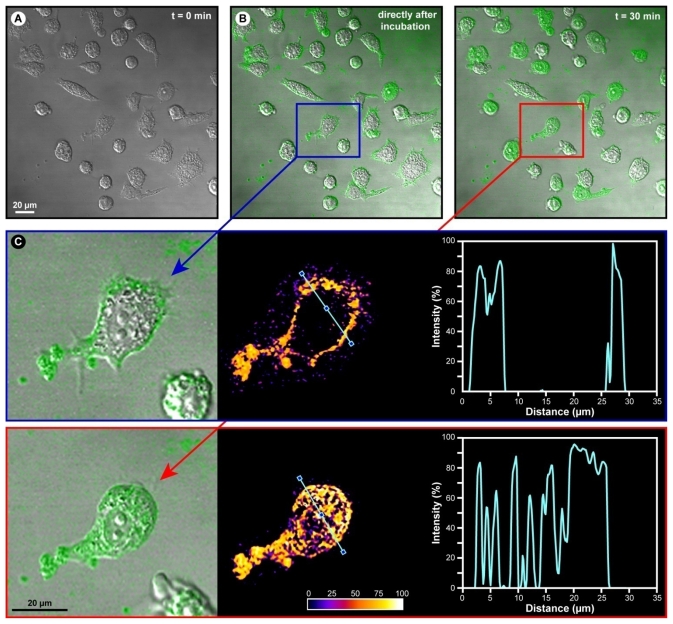
Confocal fluorescence microscopic evaluation of nanoparticle penetration and uptake in human T-cells (Jurkat cells). (**A**) Differential interference contrast image of the cell culture at t = 0 min; (**B**) DIC-fluorescence overlay: uptake evaluation, with an amplified region in (**C**): intensity gradient and intensity plot along a defined axis (blue line).

Gelatin-based nanoparticles have been shown by a number of studies to be taken up by various cell types via passive endocytic pathways. A cell culture of adhering Jurkat cells (human T-cell) was incubated with 0.5 mg/mL of GNR nanoparticles containing fluorescein isothiocyanate (FITC) as a fluorescent marker. Our confocal microscopy imaging results (Figure 10) show that initially the nanoparticles coat the cell surface of the adhering cells. However, within 30 min, the gelatin nanoparticles penetrated the T-cells and bright green FITC fluorescence was observed in the cytoplasm and other sub-cellular compartments (compare the cells in Figure 10(C)), indicating that the nanoparticles were efficiently internalized and transported within the cells. Some cells in Figure 10(B) remained unstained intracellularly, which might be cell cycle dependent. However, as the incubation time progressed, more cells became fully stained. Compared with amphiphilic gelatin nanospheres, the cellular uptake rate of GNRs is significantly faster [22]. Overall, these results show that the GNR nanoparticles are taken-up by immune cells without further need for the incorporation of targeting modalities to aid active up-take processes such as receptor-mediated endocytosis. However, future endeavors may contain such surface coatings, since this would facilitate the targeting of the GNR nanoparticles to a particular cell type of tissue within intact organisms.

## 3. Experimental Section

### 3.1. Preparation of Samples

Fresh unripe fruits of *R. coreanus* were collected from Hyeong Seuong (Gangwondo, Korea). Subsequently, the unripe fruits were shade-dried for 48 h at ambient temperature. Extracts of the *R. coreanus* fruits were made by pulverizing 50 g of shade-dried unripe fruits and extracting the water-soluble components with 1,000 mL of distilled water for 24 h at 60 °C. The solvent was evaporated with an Eyela NE-series rotary evaporator (Tokyo Rikakikai Co., Tokyo, Japan) under reduced pressure and the resulting product was freeze-dried for 24 h. The extraction yield was determined to be approximately 17%. Functional nanoparticles were produced by mixing three components: gelatin, *R. coreanus* extract, and fluorescein isothiocyanate (FITC) [47]. First, 25 mg of gelatin was dissolved with a small amount of water in a 50 mL round bottom flask and the solvent was evaporated at room temperature using a rotary evaporator to produce a dried thin gelatin film. Next, 25 mg *R. coreanus* powder was dissolved in distilled water to a final concentration of 1 mg/mL. Finally, 2 mL of HEPES (pH = 8.2) and 5 mM FITC (Sigma, St. Louis, MO, USA) were added to the aqueous extract solution of *R. coreanus* [48] and the three components were dispersed by ultrasonication with a VCX500 sonicator at 500 W (Sonics & Materials, Inc., Newtown, CT, USA) for 2 h using a rod-type CV33 probe (Sonics & Materials, Inc., Newtown, USA). The condition of the ultrasonication was fixed at 25 °C, 7:4 sec pulse to break interval, and 32% amplitude.

### 3.2. Characterization of Nanoparticles

#### 3.2.1. Transmission Electron Microscopy

The morphology of the nanoparticles was examined with a LEO-912AB TEM (LEO Electron Microscopy GmbH, Jena, Germany) operating at an accelerating voltage of 80 kV. A thin film of nanoparticles was negatively stained using phosphotungstic acid (H_3_PW_12_O_40_) and mounted on a carbon-coated grid. The grid was dried in a desiccator at room temperature (25 °C) before loading onto the microscope.

#### 3.2.2. Dynamic Light Scattering

To determine the size and size distribution of GNRs, DLS measurements were performed using a Brookhaven 90 Plus Nanoparticle Size Analyzer (Brookhaven Instruments Corp., New York, NY, USA). The intensity of the scattered light was detected at 90° to the incident beam. The light source was a 35 mW He-Ne laser emitting monochromatic light at a wavelength of 632.8 nm, which was focused onto the sample, and the scattered light was detected by a photo-multiplier tube (Hamamatsu Photonics, Hamamatsu City, Japan).

#### 3.2.3. Measurement of Zeta Potential and pH

The zeta potential of the GNRs was measured by varying the pH in a Brookhaven 90 Plus Nanoparticle Size Analyzer. About 3 mL of the suspension (1 mg/mL) was added to a cuvette and adjusted to pH values in the range from 2–10 using 0.1 N HCl or 0.1 N NaOH. The suspension was equilibrated for 4 min at 25 °C. The measurement was performed with three runs, with each run consisting of 10 single measurements.

#### 3.2.4. Encapsulation Efficiency

The oversized nanoparticles were removed by gel-permeation chromatography using Sephadex G-100 columns (1.6 cm × 40 cm; bead size 40–120 μm) purchased from GE Healthcare (Uppsala, Sweden). The collected GNR fraction was centrifuged for 30 min at 16,770 × g and the precipitate was dissolved by adding 25 mL acetone (Sigma, St. Louis, MO, USA). After 30 min of stirring, 250 mg l-cysteine (Sigma, St. Louis, MO, USA) was added. The sample was sonicated at 60 kHz for 30 min and filtered through a 0.2 μm syringe filter [49].

The content of phenolic acids in the filtrate was then determined by HPLC (M600E, M7725i/Waters, 996PDA, Waters, Milford, MA, USA). The filtrate was separated to (1) gallic acid; (2) chlorogenic acid; (3) caffeic acid; (4) ferulic acid; and (5) m-coumaric acid using a reverse-phase C18 column (250 mm × 4.6 mm, Phenomenex, Torrance, CA, USA) at 25 °C. The mobile phase consisted of 50 mM aqueous phosphoric acid solution (solvent A) and 100% acetonitrile (solvent B). The gradient elution program is shown in Table 2. The flow rate was 0.7 mL/min, UV absorbance was detected at 280 nm, and the injection volume was 20 μL for all samples (50 ppm) [16]. The encapsulation efficiency was calculated as the ratio of the peak area of the GNRs to that of the crude extract of *R. coreanus* [11] according to:

(1) $$\text{Encapsulation efficiency}(\%)=\frac{\text{amount extract nanoparticle}}{\text{amount extract added initially}}\times 100\%$$

### 3.3. Immune Activities of Nanoparticles

#### 3.3.1. *In Vitro* Cytotoxicity

The sulforhodamine B (SRB) assay was performed in HEK293 cells (Adenovirus transformed human embryonic kidney 293 cells) to investigate *in vitro* cytotoxicity. Cells were incubated in tissue culture flasks in the desired media in a humidified atmosphere at 37 °C with 5% CO_2_. Trypsinized (trypsin-EDTA, Gibco, Grand Island, NY, USA) cells were washed with media and diluted to a seeding concentration of 10^4^ cells/mL in each well of a 96 well plate. The plate was kept in the incubator for 24 h. To determine cell survival after exposure to 0.2, 0.4, 0.6, 0.8 or 1.0 mg/mL of the individual samples for 48 h, the SRB assay was performed as described previously [50] with some modification. After cultivation, 100 μL of a 20% cold trichloroacetic acid (TCA) solution was gently added on top of the medium. The plate was then incubated for 60 min at 4 °C. Wells were rinsed five times with distilled water, and then cells were stained with 0.4% SRB solution (50 μL/well) for 15 min at room temperature. The SRB staining solution was decanted and wells were rinsed five times with 1% acetic acid to remove unbound dye and left to air-dry. The bound SRB dye was then solubilized in 100 μL/well of Tris-base solution, and plates were placed on a plate shaker for 1 h at room temperature. Plates were subsequently read at 540 nm using a ThermoMax microplate reader (Molecular Devices, Sunnyvale, CA, USA), and the results were expressed as a percentage of control values [51].

#### 3.3.2*. In Vivo* Toxicity

The *in vivo* toxicity was investigated by measuring the survival rate ratio in ICR mice (traditional outbred albino mouse by Hauschka and Mirand-Roswell Park Memorial Institute, Buffalo, USA). Five-week-old female ICR mice (Orientbio Co., Ltd, Seoul, Korea) were used after 7 days of acclimatization. Mice were housed in groups of six in stainless-steel cages in a room maintained at a constant temperature (23 ± 1 °C) and humidity (60 ± 10%) under a 12 h light/dark cycle (lights on 07:30–19:30 h). Just before the experiment, the mice were only given distilled water and divided into groups fed gelatin nanoparticles (GO) or gelatin nanoparticles containing *R. coreanus* (RO) and a number of controls (Figure 5) (all 1 mg/mL). The mice were orally fed at a dosage of 1 mL/g body weight for 21 days. Every 3 days, the body weight was measured. The mice were terminated after the last bleed on day 21, and their blood was collected into 2 mL vials. The *in vivo* cytotoxicity was estimated by measuring blood glucose, cholesterol, and body weight.

#### 3.3.3. Proliferation of B- and T-Cells and Secretion of Cytokines

Raji (human B) and Jurkat (human T) cells were obtained from the American Type Culture Collection (ATCC, Manassas, VA, USA). They were maintained in RPMI-1640 supplemented with 10% fetal bovine serum (FBS, Gibco) and 100 U/mL gentamicin sulfate (Sigma) in a humidified atmosphere at 37 °C with 5% CO_2_. In six-well plates, the proliferation of human B- and T-cells was determined by direct cell counting using a hemacytometer (Hausser Scientific Company, Horsham, PA, USA) after treatment with *R. coreanus* extract or GNR (0.5 mg/mL) for 6 days [52]. Ferulic acid (4-hydroxy-3-methoxy cinnamic acid), which was reported to have immune activity [34], was used as control at the same concentration as the *R. coreanus* extract.

Secretion of cytokines was quantified by measuring the amounts of IL-6 and TNF-α with kits from Chemicon (Temecula, CA, USA). After adjusting the immune cell concentration to 1–2 × 10^4^ cells/mL, 900 μL of the cell suspension was seeded into 24-well plates and cultured for 24 h in 5% CO_2_ at 37 °C. Subsequently, 100 μL of a 0.5 mg/mL sample (*R. coreanus* extract or GNR) was added to the cells and centrifuged to obtain the supernatant, from which an absorbance reading was obtained at 450 nm using a ThermoMax microplate reader. The amounts of cytokines were determined by comparison to standards [53].

#### 3.3.4. NK Cell Proliferation

The interleukin-2 (IL-2) dependent Natural Killer Cell cell line NK-92MI, (ATCC, CRL-2408) was diluted to 2 × 10^7^ cells/mL using 2 mM L-glutamine, 0.2 mM myoinositol, 20 mM folic acid, 0.1 mM 2-mercaptoethanol, and 12.5% FBS in MEM and cultured in T-25 flasks. The proliferation was observed after each sample at a final concentration of 0.5 mg/mL. Cells were sub-cultured 3 to 4 times and centrifuged to obtain the supernatant. After 900 μL of the cell suspension was aliquoted into 24-well plates at 4–5 × 10^4^ cells/mL and allowed to adjust for 24 h, 100 μL of the supernatants from B-cells was placed into each well and the cells were cultured for 48 h. Finally, the proliferation of NK-92MI cells was observed for 6 consecutive days using a NucleoCounter NC-200 cell counter (ChemoMetec, Allerød, Denmark) [54].

#### 3.3.5. Antibody Production in Mice

Animals were challenged with GNR, GO, and controls as described under *in vivo* toxicity. IgG content was determined as follows: 96-well plates were coated overnight with 100 μL/well of an affinity-purified goat F(ab)2 anti-mouse IgG (Caltag, Burlingame, CA, USA) as primary antibody appropriately diluted in phosphate-buffered saline (PBS) at 4 °C. The wells were subsequently washed three times with PBS containing 0.05% Tween-20 (PBS/Tween) and blocked with 1% bovine serum albumin (BSA)/PBS at room temperature for 2 h. This buffer solution was also used as a diluent in all subsequent steps. After washings the blocked wells three times with PBS/Tween, 100 μL of diluted samples was. As a standard serum, a pooled mouse serum standard containing known concentrations of IgG (Pierce, Rockford, IL, USA). The plates were incubated at room temperature for 1 h before washing, as described above. Aliquots of 100 μL of horseradish peroxidase (HRP)-conjugated goat IgG (Caltag) diluted with BSA/PBS were added to each plate. The plates were further incubated for 1 h at room temperature. After washing, peroxidase activities were assayed as follows: 100 μL of substrate solution (10 mg of o-phenylenediamine and 8 μL of 30% H_2_O_2_ in 25 mL of 0.1 M citrate–phosphate buffer, pH 5) was added to each well of the plate. The plates were incubated for 15 min at room temperature, and the enzyme reaction was terminated by adding 50 μL/well of 1 N H_2_SO_4_. Optical density at 490 nm was finally measured with a microplate spectrophotometer (Sunnyvale, CA, USA).

### 3.4. Uptake of Nanoparticles by Immune Cells

To determine the uptake of nanoparticles into the immune cells, a LSM510 META NLO confocal laser scanning microscope (Carl Zeiss, Jena, Germany) was employed to image the cellular up-take process as follows: Jurkat T-cells were seeded in the confocal dish at 2 × 10^6^ cells/mL. Nanoparticles (200 μL) containing a FITC-labeled extract solution were then added to the cells. Thirty minutes later, the media of the Jurkat cells was carefully removed and the surface was washed three times with PBS buffer. The cross-sections were imaged at 543 nm using a confocal laser scanning microscope.

### 3.5. Statistical Analysis

Data are presented as the means ± SEM from at least three independent experiments. Values were evaluated by one-way ANOVA, followed by Duncan’s multiple range tests using GraphPad Prism 4.0 (GraphPad Software, Inc, La Jolla, CA, USA). Treated or untreated groups were compared using the student’s t test. Differences were considered significant at P < 0.01.

## 4. Conclusions

The preparation of gelatin nanoparticles is a common and well-established method [55]. Gelatin nanoparticles can be used as delivery vehicles in a myriad of ways, either by attaching pharmaceutical ligands to the nanoparticle’s surface [56] or by encapsulating molecules within the particle as is done for vitamin A in foods to form a physical barrier and protect it from degradation [57]. Numerous drug formulations are based on gelatin or other biopolymer particles, primarily for stabile drug delivery, to achieve a higher local concentration, and in particular formulations, a delayed or controlled release. Thus far, extracts of medicinal plants such as *R. coreanus* have received little attention in this field compared to pharmaceutical drugs. Potions made from *R. coreanus* are customarily used in traditional Asian medicine because anti-impotence, aphrodisiacal, anti-inflammatory [14], anti-bacterial [15], antioxidative [16], and even anti-tumorigenic [18] properties are ascribed to *R. coreanus*. We therefore investigated both the immunomodulatory properties of the *R. coreanus* extract and assessed the feasibility of a controlled drug delivery based on gelatin nanoparticles.

Our results demonstrate that the extract of *R. coreanus* can effectively be encapsulated in gelatin nanoparticles, which results in a relatively homogenous, well dispersed and stabile colloidal solution. Morphology evaluation via TEM showed spherical nanoparticles with an average size of 143 ± 18 nm and a bandwidth of 76 nm (FWHM) as determined by DLS. No clusters were observed, in conformity with the high negative values for the zeta potential (at physiological pH = 7.4, the average zeta potential of GNR was −19.3 mV), which ensures sufficient particle repulsion and a stabile colloidal solution. The encapsulation efficiency with nearly 60% was high compared to equivalent formulations with other drugs [21,28]. Confocal imaging with a fluorescent marker revealed that the particles initially adhered to the cell surface and that they were efficiently internalized, probably by passive mechanisms, such as endocytosis, within 30 min and subsequently homogenously dispersed within the cell.

Assessment of the toxicity showed that no significant cytotoxic effects occurred in HEK293 cell cultures, which is in agreement with previous reports that gelatin and gelatin-encapsulated nanoparticles show low toxicity and high biocompatibility [22,24,30]. Furthermore, ICR mice fed on GNR at 1 mL/g body weight showed no fatalities and no *in vivo* toxicity was observed in any of the feeding groups. Moreover, they displayed a steady weight-gain and growth, near normal glucose, reduced LDL and increased HDL cholesterol levels. These results additionally point to the potential of GNR treatment to cause health-promoting effects, although this requires further investigation.

By determining the proliferative capacity of key immune cells, *i.e.*, T-, B- and NK-cells, the secretion of cytokines, and the production of IgG immunoglobulins in ICR mice, we aimed to cover a large part of the immune response to assess the immunomodulatory effects and efficiency of the crude *R. coreanus* extract and *R. coreanus*-extract-loaded gelatin nanoparticles. As a positive control, ferulic acid (4-hydroxy-3-methoxy cinnamic acid) was used, since previous investigations report immunomodulatory properties [34].

On the whole, the crude extract induced a significant modulatory effect, which was topped by the GNR nanoparticles in all immune cells. Furthermore, the effects induced by the crude extract and the GNR nanoparticles were higher than those induced by the positive control, ferulic acid. The immune cells were not hindered in their proliferation in any way, but rather a significant increase in proliferation of a factor 2 to 3 was observed in all immune cell types for the GNR nanoparticles. Furthermore, the secretion of the key cytokines IL-6 and TNF-α by B- and T-cells was on average at a ~2–3 times higher rate compared with the extract and ferulic acid. *In vivo* immunomodulatory activity in mice fed with *R. coreanus*-gelatin nanoparticles at 1 mL/g body weight showed a ~5 times higher antibody production compared to control, a ~1.3 times higher production compared to the extract only, and a ~1.6 times higher production compared to ferulic acid. Since the HPLC analysis of the extract and GNR nanoparticles showed that a significant amount of ferulic acid is present in *R. coreanus*, it is tempting to ascribe the modulatory effects to the endogenous ferulic acid. However, the effect of the extract and GNR nanoparticles was significantly larger than the effect of ferulic acid alone and the concentration of endogenous ferulic acid in the extract was much lower than the positive control. It might therefore be entirely possible that other components, such as gallic or chlorogenic acid, synergistically enhance the effect of ferulic acid alone. This is certainly an interesting line of research to be pursued in the future. Nonetheless, the results in this paper provide some indications that the endogenous ferulic acid plays a key role in the measured immunomodulation, because previous research demonstrates glucose and LDL lowering effects [58,59], and modulation of IL-8, an inteleukin that causes local accumulation of neutrophils and regulates inflammation reactions [60].

In summary, the immunomodulatory capacity can be described as: GNR nanoparticles > crude extract (RO) ≥ ferulic acid. Furthermore, the presence of endogenous ferulic acid in *R. coreanus* must at least partially be responsible for the immune-modulation. Our results suggest that gelatin nanoparticles represent an excellent transport vehicle for *Rubus coreanus* extract and extracts from other plants generally used in traditional Asian medicine. Future endeavors will focus on targeting such gelatin nanoparticles to particular cell types or tissues to achieve a more local and effective release of active components and to minimize side-effects as much as possible. This can be achieved by coating the particle with appropriate molecules that target the particle to particular cells with particular cell surface receptors, as was done by Jain *et al.* via mannosylation of the gelatin nanoparticles, which resulted in a significantly higher uptake through the macrophage mannose-receptor in macrophages [61]. Furthermore, the nature of a number of peaks identified via HPLC and the possible cooperativity of the extract components is under investigation.

## Figures and Tables

**Table 1:** Blood component analysis of ICR mice.

	Control (mg/dL)	GO^1^ (mg/dL)	GNR^2^ (mg/dL)	RO^3^ (mg/dL)	SO^4^ (mg/dL)
**Parameter**					
Glucose	192	227	208	218	191
HDL cholesterol	49.0	51.0	72.5	54.5	47.2
LDL cholesterol	58.6	55.8	45.4	52.0	59.4

1 GO: gelatin nanoparticle-only feeding group;

2 GNR: *R. coreanus* extract gelatin nanoparticle feeding group;

3 RO: *R. coreanus* extract-only feeding group;

4 SO: Saline-only feeding group.

**Table 2:** HPLC mobile phase conditions.

Time (min)	A (%)	B (%)
0	2	98
30	15	85
60	50	50
70	98	2
75	2	98
80	2	98
